# Influence of Diets Enriched with Flavonoids (Cocoa and Hesperidin) on the Systemic Immunity of Intensively Trained and Exhausted Rats

**DOI:** 10.3390/biom12121893

**Published:** 2022-12-17

**Authors:** Patricia Ruiz-Iglesias, Malén Massot-Cladera, Francisco J. Pérez-Cano, Margarida Castell

**Affiliations:** 1 Secció de Fisiologia, Departament de Bioquímica i Fisiologia, Facultat de Farmàcia i Ciències de l’Alimentació, Universitat de Barcelona (UB), 08028 Barcelona, Spain; 2 Institut de Recerca en Nutrició i Seguretat Alimentària (INSA-UB), Universitat de Barcelona (UB), 08921 Santa Coloma de Gramenet, Spain; 3 Centro de Investigación Biomédica en Red de Fisiopatología de la Obesidad y la Nutrición (CIBEROBN), Instituto de Salud Carlos III, 28029 Madrid, Spain

**Keywords:** cocoa, exercise, exhaustion, flavanols, flavanones, immune system, lymphocytes, orange, polyphenols

## Abstract

The aim of this study was to establish the influence of flavonoid-enriched diets on the immune alterations induced by an intensive training and a final exhaustion test in rats. A flavanol-enriched diet (with 10% cocoa, C10 diet) and a flavanol and flavanone-enriched diet (C10 plus 0.5% hesperidin, CH diet) were used. Lewis rats were fed either a standard diet, C10 diet or CH diet while they were submitted to an intensive running training on a treadmill. After 6 weeks, samples were obtained 24 h after performing a regular training (T groups) and after carrying out a final exhaustion test (TE groups). The C10 diet attenuated the increase in plasma cortisol induced by exhaustion, while both the C10 and the CH diets prevented the alterations in the spleen Th cell proportion. The experimental diets also induced an increase in serum immunoglobulin concentration and an enhancement of spleen natural killer cytotoxicity, which may be beneficial in situations with a weakened immunity. Most of the effects observed in the CH groups seem to be due to the cocoa content. Overall, a dietary intervention with flavonoids enhances immune function, partially attenuating the alterations in systemic immunity induced by intensive training or exhausting exercise.

## 1. Introduction

It is well known that moderate activity exercise exerts several health benefits [1,2,3], such as enhancing immunity [1], up-regulating endogenous antioxidant enzymes [4] and reducing inflammation [2], thus decreasing the risk or improving the prognosis of several chronic diseases [3]. However, overly intense exercise may induce adverse effects on health, impairing immune function and leading to a higher risk of infections, especially those affecting the upper-respiratory tract (URTIs) and the gastrointestinal compartment [5,6]. Intensive exercise induces changes in lymphoid compartments [7,8]. In blood, exercise is followed by an intensity- and duration-dependent leukocytosis, mainly due to the mobilization of neutrophils and lymphocytes in response to a high release of catecholamines and glucocorticoids induced by exhaustion [9]. After exercise cessation, blood lymphocyte counts rapidly decrease until reaching a lymphopenia that may last up to 6 h [9], which is due to a redistribution of T helper (Th) cells in both lymphoid and non-lymphoid organs [10], as well as to a higher apoptosis among highly differentiated T cells [11]. Moreover, exercise can also modulate the function of immune cells, such as the cytotoxicity of natural killer (NK) cells, the phagocytic activity of blood phagocytes and the proliferation capacity of B and T cells, as well as their ability to secrete cytokines or produce immunoglobulins (Igs) [1,7,8,12].

Flavonoids are the most abundant polyphenols found in fruits and vegetables constituting around 75% of the total polyphenol intake in Europe [13]. They have become a subject of increasing interest because of their numerous beneficial effects on human health [14,15,16,17,18,19,20,21,22,23,24]. Their intake has been associated with lower risk of cardiovascular diseases [14], neurological disorders [15,17], type 2 diabetes [18], obesity [19] and even cancer [20,21]. In the athletic field, flavonoids and other polyphenols have been proposed as potential ergogenic aids [25], since they improve muscle function and mitochondrial biogenesis [26,27,28,29]. However, their effect on exercise performance varies among flavonoid subclasses and there is not yet enough clinical scientific evidence to draw a solid conclusion on their influence [25]. Nevertheless, because of their antioxidant [22], anti-inflammatory [23] and immunomodulatory [24] properties, their intake may be useful in the preventive management of oxidative stress [30], inflammation [31] and immune disruption [32], respectively, during intensive exercise. Flavonoids can modulate immune function and their intake is able to decrease the URTI incidence by 33% in a healthy population [33].

The most consumed flavonoid subclasses are flavanols and flavanones, mainly proanthocyanidins and hesperidin, respectively [13]. The main dietary sources of flavanols are cocoa products and green tea [13]. Cocoa flavonoid content comprises about 58% proanthocyanidins, 37% catechins, including (−)-epicatechin, (+)-catechin, (+)-gallocatechin and (−)-epigallocatechin, and, also, 4% anthocyanidins [34]. On the other hand, flavanones are found in citrus fruits and hesperidin is the most abundant, especially the 2S-hesperidin isomer [35]. Preclinical and clinical studies have associated both cocoa and hesperidin consumption with reduced oxidative stress after exercise [36,37,38,39,40,41]. Moreover, cocoa has shown interesting effects on the mucosal immunity and the gut microbiota in a rat model of acute intensive exercise, which was partially mediated by its fiber content [42]. We have also demonstrated the ergogenic and immunomodulatory effects of hesperidin supplementation in a preclinical model of exhausting exercise [40,43].

Considering this background, we hypothesize that a dietary intervention with cocoa and 2S-hesperidin could prevent the immune alterations induced by intensive training and exhausting exercise in rats, leading as well to a better exercise performance. Hence, we aimed to evaluate the effect of a cocoa-enriched diet and a cocoa- and hesperidin-enriched diet on the immune alterations present in rats submitted to a 6-week period of intensive running training and a final exhaustion test.

## 2. Materials and Methods

### 2.1. Animals

Female Lewis rats (7-week-old; Janvier Labs, Saint-Berthevin, France) were maintained at the animal facility of the Faculty of Pharmacy and Food Science at the University of Barcelona (UB). The rats were housed in polycarbonate cages, 2–3 animals per cage, under controlled conditions of temperature and humidity in a 12 h light/12 h dark cycle. Female rats were chosen because they showed a better adaptation to the treadmill and exercise performance than male rats [12,44], whereas the effects of exercise on immunological variables was not influenced by rat gender [12]. Animal procedures were approved by the Ethical Committee for Animal Experimentation (CEEA) of the UB and the Catalonia Government (CEEA/UB ref. 517/18 and DAAM 10615, respectively).

### 2.2. Exercise and Nutritional Interventions

**Figure 1:**
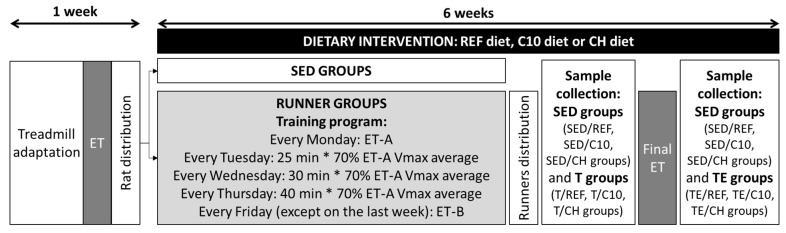
Experimental design followed in this study. ET: exhaustion test; ET-A: ET performed on Mondays; ET-B: ET performed on Fridays; REF: reference diet; C10: diet containing 10% cocoa; CH: C10 diet plus 0.5% hesperidin; SED: sedentary animals; T groups: runner groups that did not run the final ET; TE groups: runner groups that did run the final ET.

Before starting the nutritional intervention, to avoid biased distribution of animals, all rats were first familiarized with running on a treadmill (Exer3/6, Columbus, OH, USA), with an incline of 5 degrees, for one week, by increasing both the running time and the treadmill speed. Afterwards, animals performed an exhaustion test (ET) in which, after 10 min running at 18 m/min, speed was increased 3 m/min every 2 min until rat exhaustion. This was established when rats touched the shock grid more than three times (Figure 1).

Rats were then homogeneously distributed into sedentary (SED) and runner (RUN) groups with the same average running capacity, and each group was also homogeneously distributed into three groups according to the diet: reference (REF), cocoa (C10) and cocoa plus hesperidin (CH). The REF groups (SED/REF and RUN/REF groups) received the standardized maintenance diet from the American Institute of Nutrition (AIN-93M, Envigo, Huntingdon, UK). The C10 groups (SED/C10 and RUN/C10 groups) were fed an isoenergetic diet containing 10% defatted cocoa (Idilia Foods S.L., Barcelona, Spain) providing a final proportion of 3.6 g/kg polyphenols, 6.0 g/kg soluble fiber and 54.0 g/kg insoluble fiber. The CH groups (SED/CH and RUN/CH) were fed the above described C10 diet containing an additional 0.5% of 2S-hesperidin (Cardiose^®^, HealthTech BioActives, Murcia, Spain), which is the predominant isomer found in citrus fruits [35].

Once the nutritional intervention began, the runner groups (i.e., RUN/REF, RUN/C10 and RUN/CH, *n* = 18 per group) were submitted to a 6-week intensive exercise program, whereas the sedentary groups (i.e., SED/REF, SED/C10 and SED/CH, *n* = 12 per group) remained as non-exercised controls. In each week, rats carried out an ET every Monday and Friday, which consisted of running 15 min at 70% of the maximum speed average achieved in the previous Monday’s ET (the speed of the first Monday’s ET was 15 m/min), and from then on, the speed was progressively increased until exhaustion (3 m/min every 2 min). On Tuesday, Wednesday and Thursday, rats trained for 25, 30 and 40 min, respectively, at 70% of the maximum speed achieved in the previous Monday’s ET (Figure 1).

At the end of the 6-week training program, each RUN/REF, RUN/C10 and RUN/CH group was distributed into two subgroups with the same running capacity (Figure 1). The immune function was assessed at two time points: 24 h after a regular training session (named trained groups that according to the diet were: T/REF, T/C10 and T/CH groups, *n* = 9 per group), and after performing an additional final exhaustion test (named trained and exhausted groups that according to the diet were: TE/REF, TE/C10 and TE/CH groups, *n* = 9 per group) (Figure 1). The final exhaustion test consisted in running at 70% of the maximum speed average achieved in the previous Monday’s ET for 30 min and then increasing the speed progressively (3 m/min every 2 min) until exhaustion.

Throughout the study, food and water were provided ad libitum and their consumption was monitored, as well as the rats’ body weight and exercise performance.

### 2.3. Sample Collection

At the end of the study, animals were anesthetized (ketamine, 90 mg/kg, Merial Laboratories S.A., Barcelona, Spain; xylazine, 10 mg/kg, Bayer A.G., Leverkusen, Germany) and exsanguinated. Blood was immediately analyzed using an automated hematologic analyzer (Spincell, MonLab Laboratories, Barcelona, Spain). Another blood sample was used for assessing the phagocytic activity of monocytes and granulocytes. Other blood samples were used to obtain plasma and serum, which were maintained at −80 °C or −20 °C until hormone and Ig quantification, respectively. Heart, liver, thymus, spleen and gastrocnemius muscle were collected and weighed. Lymphocytes from spleen were isolated and used for characterizing the lymphocyte composition and function.

### 2.4. Plasma Cortisol and Noradrenaline Concentration

Plasma cortisol concentration was determined with the DetectX^®^ Cortisol competitive enzyme-linked immunosorbent assay (ELISA, Arbor Assays, MI, USA) following the manufacturer’s protocol. Plasma noradrenaline concentration was quantified using the Noradrenaline/Norepinephrine (NA/NE) competitive ELISA Kit (Elabscience, Houston, TX, USA) following the manufacturer’s protocol. The sensitivity of these ELISA kits was 27.6 pg/mL for cortisol and 0.19 ng/mL for NA/NE. The coefficient of variation of the intra assay precision was <14.7% for cortisol and <9.09% for NA/NE and the one for inter-assay precision was <10.9% and <10.0%, respectively. In both cases, absorbance was measured on a microplate photometer (Labsystems Multiskan, Helsinki, Finland) and data were interpolated by Ascent v.2.6 software (Thermo Fisher Scientific, Barcelona, Spain) according to the respective standard curves.

### 2.5. Spleen Lymphocyte Isolation and Phenotypic Analysis

Spleen lymphocytes were isolated in aseptic conditions by smashing the tissue in a sterile mesh cell strainer (40 µm, Thermo Fisher Scientific) as previously described [7]. After erythrocyte lysis, splenocyte numbers and viability were determined by a Countess Automated Cell Counter (Invitrogen, Thermo Fisher Scientific). Afterwards, the proportion of the different lymphocyte subsets was assessed by flow cytometry using mouse anti-rat CD161b, CD45RA, CD8α, CD4, TCRαβ or TCRγδ monoclonal antibodies (mAb) (BD Biosciences, CA, USA) conjugated either to fluorescein isothiocyanate (FITC), phycoerythrin, peridinin-chlorophyll-a protein, allophycocyanin or brilliant violet 421, as described previously described [7]. A negative control staining without mAb and a staining control for each mAb were included. Data were acquired with a Gallios™ Cytometer (Beckman Coulter, Miami, FL, USA) in the Flow Cytometry Unit (FCU) of the Scientific and Technological Centers of the UB (CCiTUB) and analyzed with FlowJo v.10 software (Tree Star, Inc., Ashland, OR, USA). The percentage of positive cells in the lymphocyte population selected was established according to forward-scatter characteristics (FSC) and side-scatter characteristics (SSC) or in a particular lymphocyte population.

### 2.6. Spleen Lymphocyte Stimulation and Proliferation

Spleen lymphocytes (5 × 10^5^ cells) were incubated in quadruplicate in 96-well plates (TPP, Sigma-Aldrich, Madrid, Spain) and stimulated or not with Concanavalin A (ConA, 5 µg/mL, Sigma-Aldrich). After 48 h, supernatants were collected and stored at −80 °C until cytokine and Ig quantifications, while T cell proliferation ability was assessed using a BrdU Cell Proliferation Assay Kit (Roche, Madrid, Spain), according to the manufacturer’s instructions. The proliferation rate was calculated by dividing the absorbance of ConA-stimulated cells by that of non-stimulated cells.

### 2.7. Cytokine and Immunoglobulin Quantification

The concentration of IgG1, IgG2a, IgG2b, IgG2c, IgM and IgA in plasma and non-stimulated splenocyte supernatants and that of interleukin (IL) 2, IL-10, interferon (IFN) γ, granulocyte colony-stimulating factor (G-CSF), granulocyte and macrophage colony-stimulating factor GM-CSF, IL-4, IL-6, IL-1α, IL-1β and tumor necrosis factor (TNF) α in ConA-stimulated splenocyte supernatants were quantified at the end of the study using ProcartaPlex^TM^ Multiplex immunoassay (Affymetrix, eBioscience, San Diego, CA, USA), according to the manufacturer’s protocol. Data were acquired by MAGPIX Cytometer (Affymetrix) in the FCU of the CCiT-UB and analysed by ProcartaPlex Analyst v1.0 software (Affymetrix). The lower limits of quantification (LLOQ) were: 1.70 ng/mL for IgG1, 1.73 ng/mL for IgG2a, 2.67 ng/mL for IgG2b, 3.67 ng/mL for IgG2c, 0.197 ng/mL for IgM, 0.584 ng/mL for IgA, 1.82 pg/mL for IL-2, 6.01 pg/mL for IL-10, 3.34 pg/mL for IFN-γ, 4.91 pg/mL for G-CSF, 4.81 pg/mL for GM-CSF, 0.62 pg/mL for IL-4, 2.19 pg/mL for IL-6, 10 pg/mL for IL-1α, 13 pg/mL for IL-1β and 2.88 pg/mL for TNFα. Total IgG was calculated as the addition of the four isotypes. In splenocyte supernatants, IgG2a, G-CSF, GM-CSF, IL-4, IL-6, IL-1α, IL-1β and TNFα levels were below the LLOQ.

### 2.8. Phagocytic Activity

The proportion and phagocytic activity of monocytes and granulocytes were quantified in blood samples using the Phagotest^TM^ kit (Glycotope, Biotechnology GmbH, Heidelberg, Germany), following the manufacturer’s instructions as previously reported [12]. Data were acquired using Gallios™ Cytometer in the FCU of the CCiTUB and the analysis of the results was carried out with FlowJo v.10 software. Monocyte and granulocyte subsets were selected according to their FSC/SSC. The proportion of FITC positive cells in each gate was considered as the percentage of monocytes and granulocytes with phagocytic ability present in the sample, whereas the mean fluorescence intensity (MFI) indicated their corresponding phagocytic activity. Changes in both blood cells’ phagocytic proportions and activities are represented considering the SED/REF group mean value as 1, therefore, all values are expressed as a fold change of the mean value with respect to the SED/REF group.

### 2.9. Natural Killer (NK) Cell Cytotoxic Activity

The cytotoxic activity of spleen NK cells was determined by the NKTEST^TM^ kit (Glycotope), according to the manufacturer’s instructions as previously described [12]. Data were acquired using a Gallios™ Cytometer (FCU in the CCiTUB) and the analysis of the results was carried out with FlowJo v.10 software. The individual cytotoxic activity was calculated according to the total NK activity and the percentage of NK cells of each sample.

### 2.10. Statistical Analysis

Statistical analysis of the data was performed using IBM Social Sciences Software Program (SPSS, version 26.0, Chicago, IL, USA) and Rstudio v4.04 (Rstudio, Inc.) with R version 3.6.1 (R Core Team 2021, R Foundation for Statistical Computing, Vienna, Austria). The normality and homoscedasticity of the data were tested by Shapiro–Wilk’s and Levene’s test, respectively. Once these conditions were confirmed, a two-way ANOVA test was applied and, if significant differences were detected, Tukey’s post hoc test was carried out. Otherwise, non-parametric aligned rank transform for non-parametric factorial ANOVA (ART-ANOVA) followed by emmeans post hoc (Tukey-adjusted *p* value) were applied, using the ARTool [45,46] and emmeans [47] packages, respectively, for Rstudio. To compare variables during the study (e.g., body weight, daily chow and water intake, and changes in distance run in the exhaustion tests), a repeated-measures ANOVA was applied. Significant differences were considered when *p* ≤ 0.05. When significant differences were detected, the *p* values obtained in the two-way ANOVA or the ART-ANOVA for the variables diet (D), exercise (E) and the interaction between them (DxE) were written in the legend box. Changes due to the dietary condition were represented with symbols in the legend box. Changes due to the exercise condition were represented in the figure using different letters above the bars. When the DxE interaction was significant, changes between groups were represented with symbols above the respective bars.

## 3. Results

### 3.1. Body Weight, Chow Intake and Training Performance

**Figure 2:**
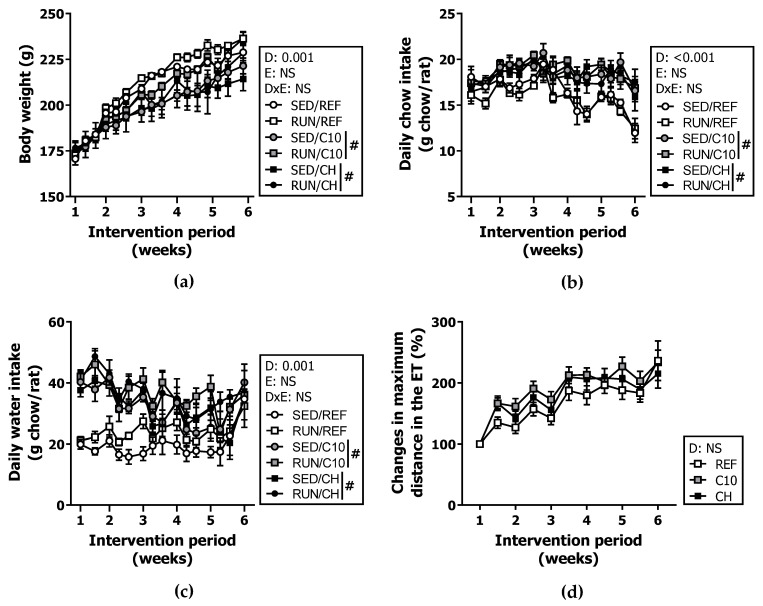
Body weight (**a**), chow intake (**b**) and water intake (**c**) throughout the 6 weeks of training and nutritional intervention, and changes in the maximum distance run in the exhaustion tests (ETs) performed throughout the study with respect to the first ET in the runner rats (**d**). Diet (D); exercise (E); diet x exercise interaction (DxE); sedentary rats (SED); runner rats (RUN); reference diet (REF); cocoa diet (C10); cocoa and hesperidin diet (CH). Data are expressed as mean ± standard error of the mean (SEM) (*n* = 6–18). Statistical differences: # *p* < 0.05 vs. REF diet; NS, no statistically significant differences detected.

Body weight (BW), chow and water intake were monitored three times per week throughout the 6 weeks of the study (Figure 2). The initial BW was similar among all the groups, but throughout the study, the animals fed C10 and CH diets, both SED and RUN groups, had a lower BW gain (time x diet interaction effect *p* = 0.001 by repeated-measures ANOVA) (Figure 2a). This effect was not associated with a lower chow intake; and on the contrary, the intake of both experimental diets was increased (*p* < 0.001) (Figure 2b). The water intake was also higher in the C10 and CH groups, which was particularly visible during the first weeks of the nutritional intervention (*p* < 0.001) (Figure 2c).

With regard to exercise performance, neither the C10 nor the CH diets modified the distance run in the weekly ETs (Figure 2d). Similarly, there were no differences in the performance of the final ET, where the RUN/REF group ran 1434.7 ± 62.48 m, the RUN/C10 group 1593.9 ± 81.00 m and the RUN/CH group 1439.9 ± 85.27 m.

**Figure 3:**
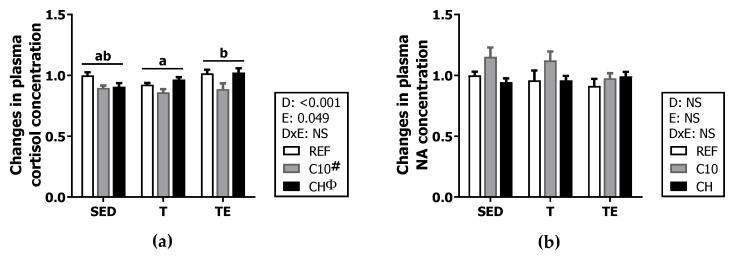
Changes in cortisol (**a**) and noradrenaline (NA) (**b**) concentration in plasma at the end of the study compared to the SED/REF group. Diet (D); exercise (E); diet x exercise interaction (DxE); sedentary rats (SED); trained rats (T); trained and exhausted rats (TE); reference diet (REF); cocoa diet (C10); cocoa and hesperidin diet (CH). Data are expressed as mean ± standard error of the mean (SEM) (*n* = 9–12). Statistical differences: *p* < 0.05 between values not sharing common letters; # *p* < 0.05 vs. REF diet; Φ *p* < 0.05 vs. C10 diet; NS, no statistically significant differences detected.

On the other hand, the C10 diet decreased plasma cortisol in the three exercise conditions (SED, T and TE) (*p* < 0.05), preventing the increase induced by the final exhaustion test when cortisol levels in the TE groups were higher than those in T groups (*p* = 0.044) (Figure 3a). Neither the exercise nor the experimental diets induced significant changes in plasma noradrenaline (Figure 3b).

### 3.2. Organ Weight

**Figure 4:**
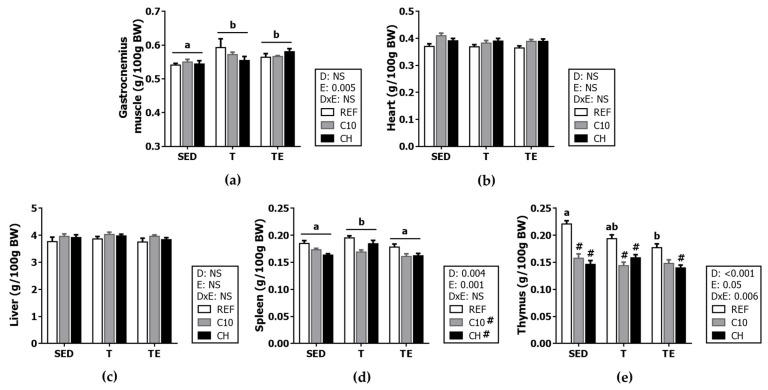
Organ relative weights at the end of the study: gastrocnemius muscle (**a**), heart (**b**), liver (**c**), spleen (**d**), and thymus (**e**). Diet (D); exercise (E); diet x exercise interaction (DxE); sedentary rats (SED); trained rats (T); trained and exhausted rats (TE); reference diet (REF); cocoa diet (C10); cocoa and hesperidin diet (CH). Data are expressed as mean ± standard error of the mean (SEM) (*n* = 9–12). Statistical differences: *p* < 0.05 between values not sharing common letters; # *p* < 0.05 vs. REF diet; NS, no statistically significant differences detected.

At the end of the study, the relative weight of the heart, liver, thymus, spleen and gastrocnemius muscle was determined (Figure 4). All exercised rats (T and TE groups) had a higher gastrocnemius muscle weight (*p* < 0.01). The relative weight of the heart and the liver was not modified by any exercise condition or the experimental diets.

With regard to immune tissues, in comparison with the respective T groups, the final exhaustion test induced a decrease in the relative weight of the spleen for all animals (*p* < 0.001). Moreover, C10 and CH diets also reduced the spleen relative weight (*p* < 0.01). On the other hand, in REF animals, the final exhaustion test decreased thymus weight with respect to the SED animals (*p* < 0.05). Additionally, the intake of the experimental diets reduced thymus weight (*p* < 0.001) in almost all conditions.

### 3.3. Hemograme

**Figure 5:**
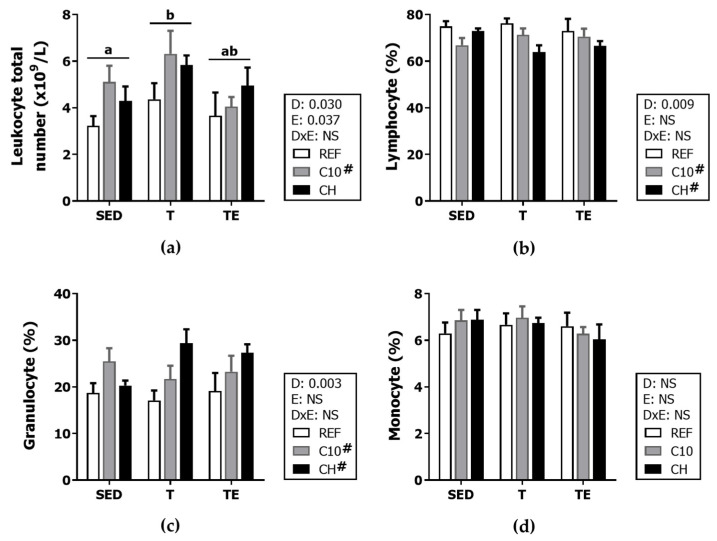
Blood counts of leukocytes (**a**) and percentage of lymphocytes (**b**), granulocytes (**c**) and monocytes (**d**) at the end of the study. Diet (D); exercise (E); diet x exercise interaction (DxE); sedentary rats (SED); trained rats (T); trained and exhausted rats (TE); reference diet (REF); cocoa diet (C10); cocoa and hesperidin diet (CH). Data are expressed as mean ± standard error of the mean (SEM) (*n* = 6–8). Statistical differences: *p* < 0.05 between values not sharing common letters; # *p* < 0.05 vs. REF diet; NS, no statistically significant differences detected.

Blood leukocyte counts were higher after the 6-week exercise training (T groups vs. SED groups) (*p* < 0.05) but not immediately after exhaustion (TE groups vs. T or SED groups) (Figure 5a) On the other hand, the consumption of the C10 diet increased the number of circulating leukocytes (*p* < 0.05) (Figure 4a). This increase seems to be due to a higher proportion of granulocytes (*p* < 0.05), since the lymphocyte and monocyte proportions decreased or remained unchanged, after the intake of the C10 diet (Figure 5b–d). A significant increase in granulocyte proportion was also observed after the intervention with the CH diet (*p* < 0.01) (Figure 5c).

**Figure 6:**
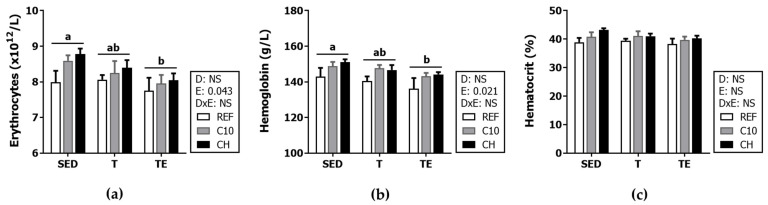
Blood counts of erythrocytes (**a**); hemoglobin concentration (**b**); and hematocrit (**c**) at the end of the study. Diet (D); exercise (E); diet x exercise interaction (DxE); sedentary rats (SED); trained rats (T); trained and exhausted rats (TE); reference diet (REF); cocoa diet (C10); cocoa and hesperidin diet (CH). Data are expressed as mean ± standard error of the mean (SEM) (*n* = 6–8). Statistical differences: *p* < 0.05 between values not sharing common letters; NS, no statistically significant differences detected.

With regard to red blood cell variables, the erythrocyte counts and the hemoglobin concentration decreased immediately after the final exhaustion (TE groups vs. SED groups) in all dietary conditions (*p* < 0.05) (Figure 6). The experimental diets did not significantly modify these variables. Neither exercise nor the diets significantly modified the hematocrit.

### 3.4. Serum Immunoglobulins

**Figure 7:**
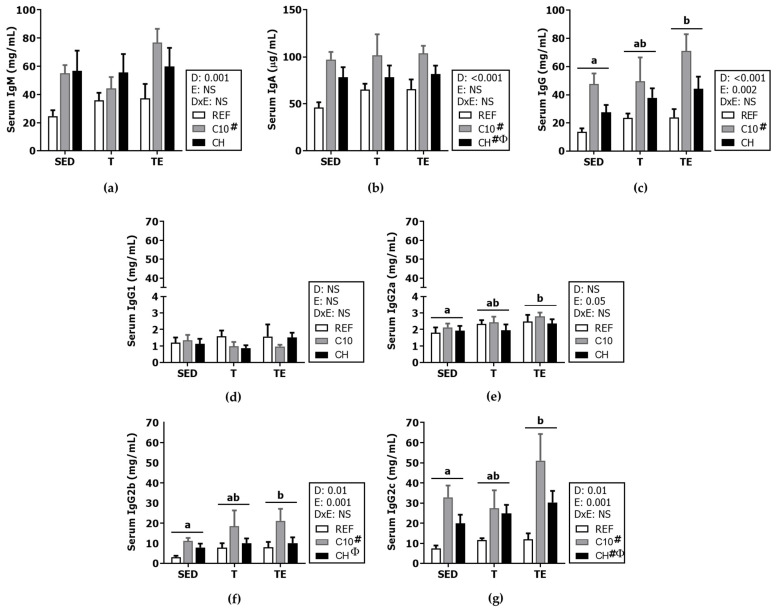
Immunoglobulins (Ig) concentration in serum at the end of the study: IgM (**a**), IgA (**b**), IgG (**c**), IgG1 (**d**), IgG2a (**e**), IgG2b (**f**), IgG2c (**g**). Diet (D); exercise (E); diet x exercise interaction (DxE); sedentary rats (SED); trained rats (T); trained and exhausted rats (TE); reference diet (REF); cocoa diet (C10); cocoa and hesperidin diet (CH). Data are expressed as mean ± standard error of the mean (SEM) (*n* = 9–12). Statistical differences: *p* < 0.05 between values not sharing common letters; # *p* < 0.05 vs. REF diet; Φ *p* < 0.05 vs. C10 diet; NS, no statistically significant differences detected.

Neither the exercise training nor the final exhaustion test modified the serum concentration of IgM and IgA (Figure 7a,b). However, the C10 and the CH diets increased the serum IgA concentration (*p* < 0.001), and the C10 diet also raised that of IgM (*p* < 0.05).

The serum IgG concentration was higher after the final exhaustion (TE groups vs. SED groups, *p* < 0.01) (Figure 7c), mainly due to increases in the concentration of IgG2b and IgG2c isotypes (*p* < 0.001) (Figure 7f,g), and a minor increase in IgG2a levels (Figure 7e). Moreover, animals fed the C10 diet, showed threefold higher serum IgG2b and IgG2c content (*p* < 0.01).

### 3.5. Phagocytic Activity

**Figure 8:**
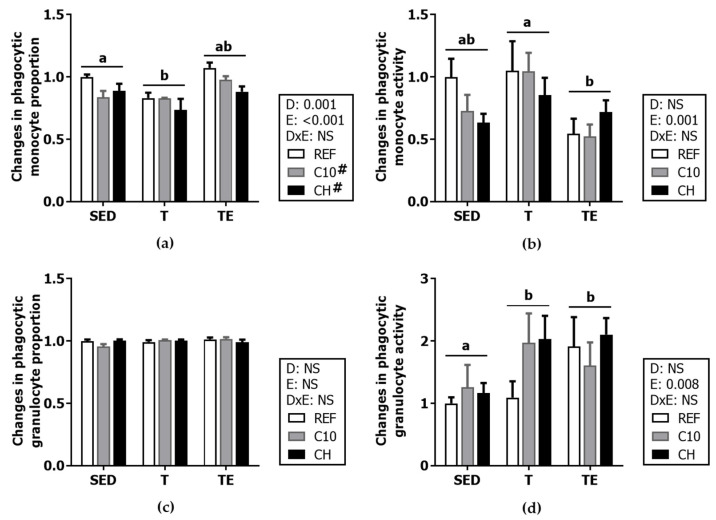
Changes in blood phagocytic monocyte proportion (**a**); blood monocyte phagocytic activity (**b**); blood phagocytic granulocyte proportion (**c**); and blood granulocyte phagocytic activity (**d**) compared to the SED/REF group. Diet (D); exercise (E); diet x exercise interaction (DxE); sedentary rats (SED); trained rats (T); trained and exhausted rats (TE); reference diet (REF); cocoa diet (C10); cocoa and hesperidin diet (CH). Data are expressed as mean ± standard error of the mean (SEM) (*n* = 6–8). Statistical differences: *p* < 0.05 between values not sharing common letters; # *p* < 0.05 vs. REF diet; NS, no statistically significant differences detected.

The proportion of phagocytic monocytes and granulocytes and their phagocytic activity were quantified in blood (Figure 8). The proportion of phagocytic monocytes in SED/REF animals was 63.88 ± 4.35% (with respect to total monocytes) and 93.42 ± 1.24% for phagocytic granulocytes (with respect to total granulocytes).

The training decreased the monocyte proportion (T groups vs. SED groups, *p* < 0.005), and the final exhaustion reduced their phagocytic activity (TE groups vs. T groups, *p* < 0.05). Both the C10 and the CH diets decreased the monocyte proportion (*p* < 0.05) without significantly affecting their functionality.

No changes in the proportion of phagocytic granulocytes were observed due to exercise or the experimental diets. However, their phagocytic activity was increased by training and the final exhaustion test (T and TE groups vs. SED groups, *p* < 0.05).

### 3.6. Spleen Lymphocyte Composition and Function

**Figure 9:**
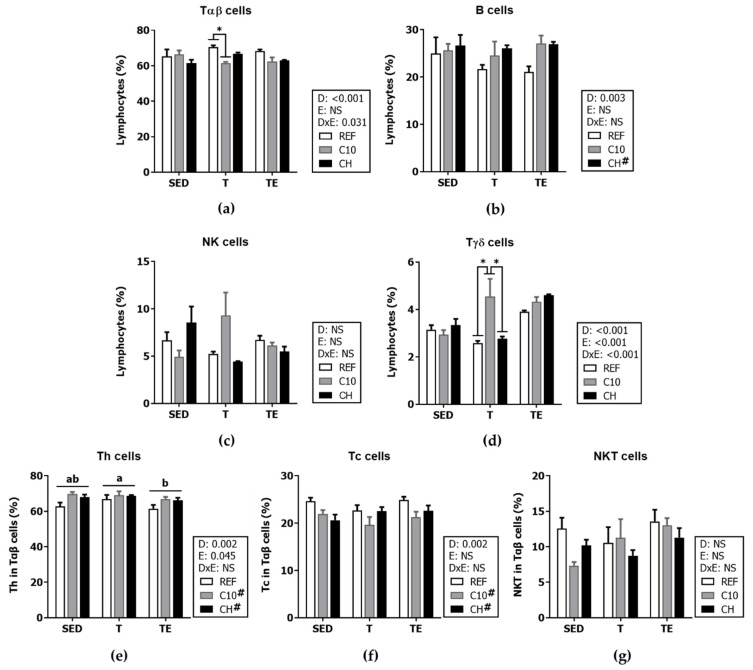
Percentage of spleen lymphocytes: T (TCRαβ+) (**a**); B (CD45RA+) (**b**); natural killer (NK) (CD161b+ TCRαβ−) (**c**); Tγδ (TCRγδ+) (**d**); Th (CD4+CD161b− in TCRαβ+) (**e**); Tc (CD8+CD161b− in TCRαβ+) (**f**); and NKT (CD161b+ in TCRαβ+) (**g**) cells. Diet (D); exercise (E); diet x exercise interaction (DxE); sedentary rats (SED); trained rats (T); trained and exhausted rats (TE); reference diet (REF); cocoa diet (C10); cocoa and hesperidin diet (CH). Data are expressed as mean ± standard error of the mean (SEM) (*n* = 6–8). Statistical differences: *p* < 0.05 between values not sharing common letters; * *p* < 0.05; # *p* < 0.05 vs. REF diet; NS, no statistically significant differences detected.

The training did not modify the major lymphocyte populations’ Tαβ cells, B cells, NK cells or Tγδ cells in animals fed the REF diet (Figure 9). However, the C10 diet decreased Tαβ cell proportion in the T group (*p* < 0.001) while the CH diet increased that of B cells in all exercise conditions (*p* < 0.01). With regard to the percentage of Tγδ cells, trained rats fed the C10 diet showed a higher percentage of Tγδ cells than those fed REF or CH diets (*p* < 0.001).

With regard to the proportion of Th and Tc cells in Tαβ cells, Th cell percentage decreased after exhaustion with respect to trained conditions (the TE groups vs. T groups, *p* < 0.05) but both the C10 and the CH groups showed higher Th cell proportions than the REF groups (*p* < 0.01). The experimental diets reciprocally decreased the proportion of Tc cells (*p* < 0.01), without modifying that of NKT cells.

**Figure 10:**
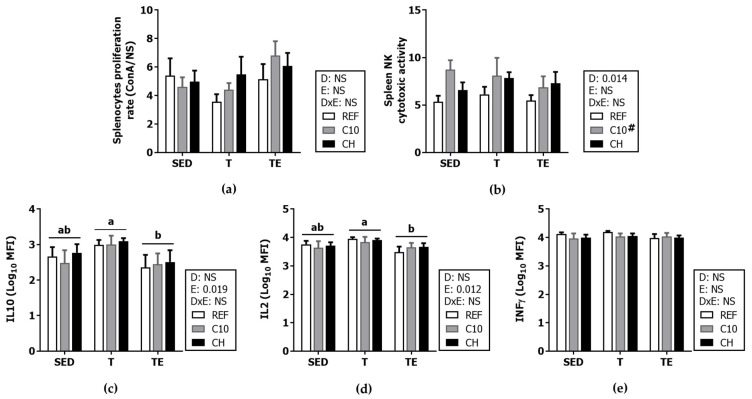
Spleen lymphocyte functionality: proliferation response (**a**), NK cytotoxicity (number of dead target cells per 100 effector cells) (**b**), and cytokine release under concanavalin A (ConA) stimulation (**c**–**e**). Diet (D); exercise (E), diet x exercise interaction (DxE); sedentary rats (SED); trained rats (T); trained and exhausted rats (TE); reference diet (REF); cocoa diet (C10); cocoa and hesperidin diet (CH). Data are expressed as mean ± standard error of the mean (SEM) (*n* = 6–8). Statistical differences: *p* < 0.05 between values not sharing common letters; # *p* < 0.05 vs. REF diet; NS, no statistically significant differences detected.

**Figure 11:**
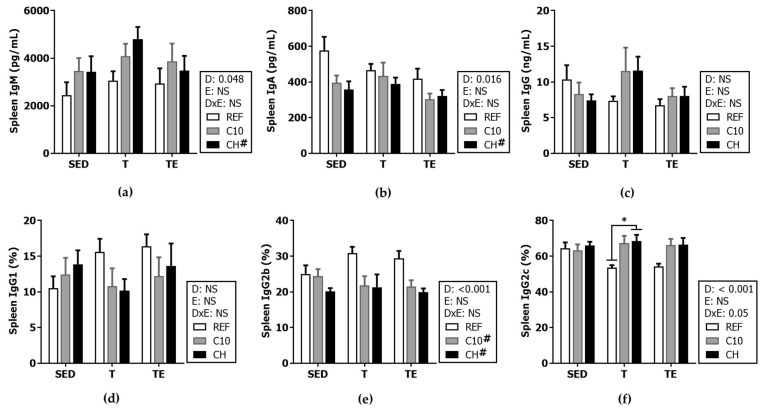
Immunoglobulins concentration in spleen lymphocyte supernatants: IgM (**a**), IgA (**b**), IgG (**c**), IgG1 (**d**), IgG2b (**e**), and IgG2c (**f**). The IgG subclasses (**d**–**f**) were calculated as the percentage of each particular isotype concentration with respect to the total IgG concentration. Diet (D); exercise (E), diet x exercise interaction (DxE); sedentary rats (SED); trained rats (T); trained and exhausted rats (TE); reference diet (REF); cocoa diet (C10); cocoa and hesperidin diet (CH). Data are expressed as mean ± standard error of the mean (SEM) (*n* = 6–8). Statistical differences: * *p* < 0.05; # *p* < 0.05 vs. REF diet; NS, no statistically significant differences detected.

The impact of exercise and the nutritional interventions on the function of spleen lymphocytes was also studied (Figure 10 and Figure 11). The splenocytes proliferation ability was similar among all the groups (Figure 10a), while the cytotoxicity of NK cells was higher in those fed C10 diet (*p* < 0.05) (Figure 10b).

The splenocyte cytokine secretion capacity after in vitro stimulation was also assessed (Figure 10c–e). In SED/REF animals, splenocytes secreted 625.7 ± 316.31 pg/mL of IL-10, 1112.9 ± 354.18 pg/mL of IL-2, and 9337.7 ± 1564.15 pg/mL of IFN-γ. The cells collected after the final exhaustion test (TE groups) produced lower levels of IL-10 and IL-2 than trained groups (*p* < 0.05). None of the experimental diets modified the secretion of the studied cytokines.

Finally, the in vitro ability for Ig production was assessed in supernatants from spleen lymphocytes (Figure 11). Neither training nor the final ET modified the in vitro secretion of the Ig by splenocytes. Both the C10 and the CH diets decreased the proportion of IgG2b (*p* < 0.01) (Figure 11e). The CH diet increased the concentration of IgM and lowered that of IgA (*p* < 0.05 in both cases), while the proportion of IgG2c in the T group also increased (*p* < 0.05).

## 4. Discussion

We have evaluated the effect of a chronic and intensive training (T group) and an additional exhaustion test (TE group) on some biomarkers of the immune system. We have also assessed the influence of a diet enriched in flavanols (10% cocoa diet, C10 diet) and a diet enriched in flavanols and a flavanone (C10 diet with 0.5% hesperidin, CH diet) on these biomarkers. We observed some changes induced by training, such as a higher granulocyte phagocytic activity, and other changes produced by the additional exhaustion test, such as a decreased blood monocyte phagocytic activity and a lower Th cell percentage in the spleen. When considering the effects of the C10 and CH diets on the changes in the immune biomarkers, we observed that both diets prevented the Th cell proportion decrease induced by the exhaustion test. Moreover, the diets had other effects beyond those induced by exercise: both diets decreased the proportion of phagocytic monocytes, and the C10 diet induced a higher cytotoxic activity for spleen NK cells. The C10 diet also induced higher serum concentrations of IgM, IgA and IgG, particularly of those isotypes linked to Th1 activity in rats [48], activity that is involved in fighting against intracellular pathogens.

Firstly, considering the exercise performance, we did not observe an improvement after 6 weeks of nutritional interventions with cocoa or cocoa plus hesperidin. This result agrees with most of the preclinical and clinical studies involving cocoa products, such as cocoa powder [38,42,49], cocoa flavanol capsules [37] or chocolate [36,50,51]. With regard to hesperidin, both preclinical [40,41,43] and clinical studies [35,39,52,53] have reported its ergogenic effects. The dose of the flavanone used in such preclinical studies was about 600–700 mg/kg per week [40,43]. In the current study, in terms of the average daily rat food intake, the diet we used meant a consumption of 3150 mg/kg BW of hesperidin per week, which was much higher than that provided in the studies that supplemented by oral gavage [40,41,43]. The lack of an ergogenic effect of the CH diet may be due to some interaction between hesperidin and the cocoa components, as well as the fact that rats took the hesperidin in the food throughout the day, meaning a slower intake rate. Further studies should elucidate the importance of the method of administration of hesperidin or other flavonoids alone to achieve an ergogenic effect.

The spleen is the largest secondary lymphoid organ in the body and plays an essential role in maintaining immune homeostasis. We observed that six weeks of intensive training induced a higher relative spleen weight with no changes in the main spleen lymphocyte subsets. These results agree with a similar study, where intensive training did not alter spleen lymphocytic proportions [43]. After the additional exhaustion test, spleen relative weight decreased, and there were not changes in the percentages of Tαβ, B and NK cells, but the proportion of the Th cell subset decreased. This may be due to Th cell mobilization to the blood [9]. These changes were accompanied by a decrease in the secretion of IL-10 and IL-2 of these cells under stimulation, but we did not find a lower proliferative activity. The lower proportion of Th cells induced by exhaustion was prevented by the intake of the C10 and CH diets. This effect could probably be attributed to cocoa, since cocoa intake in non-exercised rats induced a similar effect that could be due to an enhancing effect of cocoa on thymus Th cell maturation [54]. On the other hand, trained rats fed C10 diet had a lower proportion of Tαβ cells and a higher one of Tγδ cells, as previously observed in the mesenteric lymph nodes of non-exercised rats [55]. The increase of the Tγδ cell proportion in the later study was suggested to be due to a possible migration from the intraepithelial intestinal compartment to mesenteric lymph nodes. A further phenotypic study of Tγδ cells could shed some light in this aspect. On the other hand, the CH diet induced a higher proportion of B cells, which may be attributed to both cocoa and hesperidin, since the C10 groups also tended to have higher levels of B cells. With regard to spleen NK cells, although neither exercise nor the diets modified their proportion, the C10 diet enhanced their cytotoxic activity in all exercise conditions. The effect of cocoa on NK activity could be related to its antioxidant effect, since oxidative stress seems to reduce the expression of the activating receptor NKG2D in NK cells [56].

After 6 weeks of intensive exercise, trained rats had a higher number of blood leukocytes and, after exhaustion, there was a decrease in the erythrocyte counts and hemoglobin concentration, which could be due to the hemodilution induced by plasma volume expansion in the recovery period [57]. None of the experimental diets prevented the changes in red and white blood cells. Indeed, animals fed the C10 diet had even higher leukocyte counts than those fed REF diet, which was due to a higher granulocyte proportion. These results agree with the results obtained in a clinical study about acute exercise [58] and could be due to a lower granulocyte migration to tissues due to an effect of cocoa flavanols on adhesion molecules as reported [59].

On the other hand, the final exhaustion restored the increased leukocyte counts achieved in trained rats. It is well documented that a single bout of intensive exercise is followed by an immediate increase in blood leukocytes which has been attributed to noradrenaline release [9]. Here, exhausted rats did not show increased plasma noradrenaline concentration, although they did have higher plasma cortisol levels. This must be due to the fact that the sympathetic nervous system activation during exercise precedes that of the hypothalamic–pituitary–adrenal axis and cortisol release [9]. In fact, some studies report the decrease in catecholamine increased levels after 10 and 60 min of intensive exercise [60,61]. These studies denote the importance of the time of sampling. Here, although we aimed to assess the changes immediately after exercise cessation, technical limitations meant that blood collection was actually performed 20–30 min after extenuation, which may be enough to normalize the number of circulating leukocytes and the concentration of plasma noradrenaline. On the other hand, the cocoa diet attenuated the cortisol increase, which is in line with previous studies that attribute such effect to the inhibition 11β-hydroxysteroid dehydrogenase type 1 activity by cocoa flavanoles [38,62].

Exercise and the experimental diets affected the blood phagocytic activity. Training and exhaustion increased granulocyte phagocytic activity, although exhaustion decreased that of blood monocytes. The higher phagocytic granulocyte activity agrees with a study performed in mice, in which an increased bronchoalveolar macrophages phagocytic capacity was found after running to exhaustion on a treadmill [63]. This must be due to the release of glucocorticoids and catecholamines induced by intensive exercise [64]. None of the experimental diets influenced the phagocytic activity, although they did lower the proportion of monocytes with phagocytic capacity. These effects may be attributed to the cocoa consumption because it has been reported that cocoa reduces the expression of scavenger receptors and adhesion molecules such as the integrin VLA-4 and L-selectin, as well as that of CD40 and CD36 [65].

With regard to serum immunoglobulins, the final exhaustion test increased IgG concentration, which is consistent with both preclinical [7] and clinical studies [66,67,68]. This could be explained by the longer IgG half-life [66] observed after the chronic practice of moderate intensity exercise. The consumption of the C10 diet increased serum IgG, IgM and IgA concentrations, which was quite surprising because in previous preclinical studies no changes [69] or even decreased levels [70] were found in these Igs. The controversy among these results may be caused by the use of different rat strains, ages or sexes, as well as different cocoa batches containing different theobromine content, which seems to be the main component responsible for the effect of cocoa on antibody levels [54]. With regard to the different IgG isotypes, the final exhaustion test induced a higher concentration of IgG2a, IgG2b and IgG2c. Furthermore, the intake of the experimental diets, especially the C10 diet, increased the levels of those isotypes linked to the Th1 antibody response in rats, isotypes that promote the cytotoxic activity against infected cells, for example, by viruses. A previous study using rats of a similar age also found higher serum levels of IgG2c after a dietary intervention with cocoa [71], although another study using younger animals found the opposite effect [54], denoting the importance of age when assessing changes on immune function. In any case, our results from the C10 diet found here highlight the importance of this diet potentially counteracting or reducing the viral infections that could be induced by a higher intensity exercise and exhaustion. On the other hand, both the C10 and the CH diets enhanced serum IgA levels, which help to increase protection against potential viral infections, such as URTIs, whose risk is increased in athletes [5,6]. Further studies may clarify the real impact of these changes, assessing their potential protective effect after the induction of a viral infectious process.

With regard to the in vitro production of immunoglobulins, there was no effect of exercise, but the CH diet increased the IgM secretion, which could be associated with the higher spleen proportion of B cells observed in these animals. Neither exercise nor the diets modified the IgG in splenocyte supernatants, with the exception of a decrease in the IgG2b isotype production induced by the experimental diets.

## 5. Conclusions

Overall, our results evidence some immune changes in intensively trained Lewis rats, such as a higher granulocyte phagocytic activity. In addition, when a final exhaustion test was performed, a lower Th cell percentage in the spleen were found, whereas high granulocyte phagocytic activity was maintained. Diets containing 10% cocoa, with or without 0.5% hesperidin, prevented the alterations in Th cell percentage induced by exhaustion and produced changes in the spleen lymphocyte proportions and functions beyond those effects of exercise.

## Data Availability

Data is contained within the article.
